# Analysis and implementation of the DynDiff tool when comparing versions of ontology

**DOI:** 10.1186/s13326-023-00295-7

**Published:** 2023-09-28

**Authors:** Sara Diaz Benavides, Silvio D. Cardoso, Marcos Da Silveira, Cédric Pruski

**Affiliations:** 1 Luxembourg Institute of Science and Technology, 5, avenue des Hauts-Fourneaux, L-4362 Esch-sur-Alzette, Luxembourg; 2 Dynaccurate, 9, avenue des Hauts-Fourneaux, L-4362 Esch-sur-Alzette, Luxembourg

**Keywords:** Diff computation, Ontology management, Ontology evolution, Knowledge graph evolution

## Abstract

**Background:**

Ontologies play a key role in the management of medical knowledge because they have the properties to support a wide range of knowledge-intensive tasks. The dynamic nature of knowledge requires frequent changes to the ontologies to keep them up-to-date. The challenge is to understand and manage these changes and their impact on depending systems well in order to handle the growing volume of data annotated with ontologies and the limited documentation describing the changes.

**Methods:**

We present a method to detect and characterize the changes occurring between different versions of an ontology together with an ontology of changes entitled DynDiffOnto, designed according to Semantic Web best practices and FAIR principles. We further describe the implementation of the method and the evaluation of the tool with different ontologies from the biomedical domain (i.e. ICD9-CM, MeSH, NCIt, SNOMEDCT, GO, IOBC and CIDO), showing its performance in terms of time execution and capacity to classify ontological changes, compared with other state-of-the-art approaches.

**Results:**

The experiments show a top-level performance of DynDiff for large ontologies and a good performance for smaller ones, with respect to execution time and capability to identify complex changes. In this paper, we further highlight the impact of ontology matchers on the diff computation and the possibility to parameterize the matcher in DynDiff, enabling the possibility of benefits from state-of-the-art matchers.

**Conclusion:**

DynDiff is an efficient tool to compute differences between ontology versions and classify these differences according to DynDiffOnto concepts. This work also contributes to a better understanding of ontological changes through DynDiffOnto, which was designed to express the semantics of the changes between versions of an ontology and can be used to document the evolution of an ontology.

## Background

Annotated datasets are key for the successful implementation of Artificial Intelligence systems. These annotations, whether generated manually or automatically, are usually linked to controlled vocabularies or ontologies to explicitly express a common understanding of the annotated data, making them interpretable by humans and machines, and thus promoting more efficient reuse by algorithms. For instance, medical ontologies play a key role in data-intensive tasks, such as reporting on diagnoses, medical procedures, medications, body characteristics, etc. and are widely used by healthcare professionals and institutions involved in providing health services to patients [1–4]. It is therefore important to keep the content of these termino-ontological resources up-to-date, especially for dynamic domains such as life sciences. More than 1000 biomedical ontologies are available in public repositories such as Bioportal [5] and they change over time. Implementing a fully manual documentation process is not realistic, since the changes need to be understandable by humans and computers. Therefore, there is a need to efficiently and automatically document the changes in the ontologies as well as to determine the impact of these changes in dependent resources such as semantic mappings and annotations [6]. This chain of maintenance process is necessary to ensure interoperability between systems and understandability of documents.

In our previous work, we studied the impact of ontology changes on associated mappings [7] and semantic annotations [8]. Based on our findings, we have designed rule-based methods to (semi-)automatically maintain mappings [9] and annotations [10] rendered invalid/outdated by the evolution of underlying ontologies. These rules require, as input, the type of change occurring in the underlying ontology since it will conditioned the mapping adaptation (or annotation adaption) actions to be applied to maintain the validity of existing mappings and semantic annotation. For instance, detecting that a concept has been split into other concepts in the new version of an ontology will not trigger the same mapping adaptation action as if the concept was deleted. The mappings impacted by the split could, for instance, be associated to the new concepts (resulting from the split), while the later case (concept delete) will result into mapping delete. This is why, we need to identify and characterize the changes that occurred between two ontology versions [11–13]. This problem, known as the *Diff problem*, has already been approached in different ways in the literature. Logging or *structural diff* [14–16] consists of using a log file, often generated manually by experts, where changes are stored incrementally. The file is updated every time a new modification takes place, and the changes can be searched for using temporal queries. However, this manual work is error-prone and an automatic log generator that calculates the changes *a posteriori* by comparing two input versions of an ontology was proposed. This solution solved some inconsistencies detected in the manual log generation process. One of the first implementations of this type of approach was PromptDiff [11], in which changes are computed between two versions of an ontology using a set of rules. The identified changes are presented as instances of the Changes and Annotation Ontology (ChAO) [17]. ChAO was designed to facilitate collaborations between humans. It supports the generation of annotations about users’ actions e.g. whether a change was accepted (and some comments about reasons for the decision). Thus, a lot of useful information was expressed in the comments, and the types of changes were limited to just a few classes. This format was widely adopted by Protégé users, however, the semantics of changes remains difficult to exploit since there is no consensus on a standard description language for the changes. Another type of approach uses ontology axioms to detect the consequences of the changes on the semantics of the new version of the ontology [13, 18, 19]. The resulting changes are complementary to the previous approaches, but they are still not intuitive for end-users since they are expressed as Description Logics rules. The size of the ontologies of the biomedical domain and the frequency of changes demand more advanced solutions for the Diff calculation. COnto-Diff (Complex Ontology Diff) [12] is a tool that proposes a two-phase *diff* approach: during the first phase, there is a matching algorithm to determine conceptual mappings between versions. The second phase generates high-level changes like merge and split of concepts based on these mappings. COnto-Diff was evaluated with biomedical ontologies expressed in OWL and OBO, demonstrating the efficiency of the tool to detect changes in these languages. Another relevant approach is presented by Papavasileiou et al. [20]. Their work was designed to compute the diff between RDFS datasets. The authors propose a language of changes with 132 change actions, complete application semantics and the proof of completeness, consistency, uniqueness and non-overlapping classes. Their algorithm starts by computing the delta of triples to identify all triples that change, i.e. added and deleted triples, and regroups these triples to compose more human representative changes (Basic, Composite and Heuristic Changes). The approach prevents triples being re-used to compose more than one type of change. One of the innovative aspects of this language is that it takes into account the schema (i.e. classes and properties), instance level (i.e. individuals) and group of these schema entities (i.e. meta-classes and meta-properties). These five groups allow to focus on the nodes rather than the edges of the graph and they consider that this makes the language more human-intuitive. The approach was designed to be applied to RDFS, limiting its use to more sophisticated axioms like those provided by OWL. The *matcher* is an optional feature to map the changes and establish potential semantic links between them. In a more recent work [21], the authors provide a unified list of complex changes together with a classification for those complex changes. In [22], the method introduced focuses on RDF data (not on ontologies) to detect and express elementary and complex changes. Similarly, in [23] the authors proposed a declarative language for defining complex changes on RDF(S) knowledge bases and discussed how this language can be used to detect complex change instances among dataset versions (which can be queried for analysing evolution).

In our work, we cover OWL ontologies that have concepts, relationships, individuals and properties. The approaches of [12, 20] inspired us to design *DynDiff*^1^ for the formal description of types of changes and detection rules; DynDiff has the following characteristics:

- Rule-based system: It is based on specific change detection rules. This set of rules constitutes change patterns allowing the classification of the changed entities into different categories. The rules were defined in a revision process of the state-of-the art approaches, aiming to combine the strongest points from existing approaches [12, 20] and extend them.
- Human and machine-interpretable: The classification of changes provided by our approach is intuitive for humans and, at the same time, granular enough to be used by machines. This classification is represented as an ontology of changes (DynDiffOnto) and is made available to the community at <https://git.list.lu/dynaccurate/change-ontology>. The proposed ontology was designed following the best practices of the Semantic Web [25] and the FAIR principles [26].
- Decoupled from heuristic semantics: End-users have the possibility of configuring the matcher used to compute some types of changes. This option allows a detachment from heuristic strategies that add a randomness to the results and allows end-users to benefit from the latest advances in the domain without changing the DynDiff code.
- Scalable and robust: The solution is able to work with different sizes of inputs i.e. ontologies with a small or large number of ontological elements. The outputs (i.e. change types) are computed in an efficient manner. Our experiments show that DynDiff is able to compute Diffs between large ontologies in a reasonable time (less than 1,000 sec to compute more than 70,000 changes)

Based on these characteristics, we made the following contributions [24]:

- A change language that extends those proposed by Hartung et al. [12] and Papavasileiou et al. [20] by proposing rules for individuals and making the distinction between relationships (e.g. addSubGraph) and attributes (e.g. we distinguish between comment, label and other attributes). As evoked, many tools are restricted to classes only, limiting the exploitation of the changes in instances. The proposed change language is presented as an ontology of changes (DynDiffOnto) and the instantiation of the classes of this ontology represents the changes that were detected between the different versions. Inspired by previous attempts using ontologies to specify changes [27], the objective of this ontology is to give a way to document the changes at ontology evolution time which will therefore be unambiguously interpreted by user exploiting the newly release version of these dynamic ontology.
- A change detection algorithm that provides a classification of changes based on DynDiffOnto classes. This structure provides transparency for the user in terms of the description of the results.

In this paper, we i) refine the explanation of the previous contributions, ii) give further details of the construction of the DynDiffOnto and iii) evaluate the proposed framework on a set of biomedical ontologies taking into account to several dimensions, such as time of execution, influence of the selected ontology matchers and the capacity of DynDiff to classify identified ontological changes.

## Methods

Our objective is to design scalable methods for computing the Diff between ontology versions, including the diff between classes, properties and individuals. By scalable, we mean an algorithm able to analyse small and large ontologies, such as SNOMEDCT or NCI thesaurus. In this section, we will formalize the problem we are addressing and detail the solution we have designed.

### Problem statement

Ontology Diff approaches look for a set of rules or heuristics that enable the detection and description of changes between two versions of the same ontology. It is a function Diff($O_{old}$,$O_{new}$) that takes two ontology versions as input and produce a set of triples as output. In our work, an ontology is a set of RDF triples [20]

$$\begin{aligned} \mathcal {T}=\textbf{U} \times \textbf{U} \times (\textbf{U} \cup \textbf{L}) \end{aligned}$$

where $\textbf{U}$ denotes resources (all things described by RDF) and $\textbf{L}$ denotes literals (values such as strings and integers). The general problem addressed in our work can be described as follows:

Given two versions of an ontology ($O_{old}$ and $O_{new}$), we aim to:

1. Determine the set of all **b**asic **c**hanges *BC* computed according to the definition given by [20]:
  1 $$\begin{aligned} BC =\{t\in \mathcal {T}|t \in (O_{new} \setminus O_{old} ) \quad \text {or} \quad t \in (O_{old} \setminus O_{new} )\} \end{aligned}$$
2. Determine the sets of **c**omplex **c**hanges *CC* and **h**euristic **c**hanges *HC*, where *CC* and *HC* are disjointed, i.e. $CC \cap HC = \emptyset$ and *HC* and *CC* form a partition of *BC*.
3. Determine the subsets of **b**asic **c**hanges $BC^*$ that have all basic changes that were not used to compose neither *HC* nor *CC*.

### The DynDiffOnto ontology

The DynDiff framework presented in this paper aims to identify changes between two ontology versions. These changes are formalized as concepts of the DynDiffOnto. The idea of having an ontology to formalize the change is to provide a mean to unambiguously interpret the type of change that DynDiff is able to detect. This will help people in charge of generating new versions of ontology to document the changes in an unambiguous format that can later be interpreted by user/systems exploiting their ontology as it is done in [9, 28]. This ontology could in the future be extended with other type of changes and could also be reused by other diff computation approaches to have a unified way of representing ontological changes. It has been designed based on the NeON methodology for ontology building [25] and the FAIR principles to optimize findability, accessibility, interoperability and reuse [26]. We have used the well-known Protégé ontology editor^2^ and the OWL2.0 W3C recommendation and in particular the RL profile, by virtue of its abilities to represent advanced axioms and its support for reasoning.

**Fig. 1:**
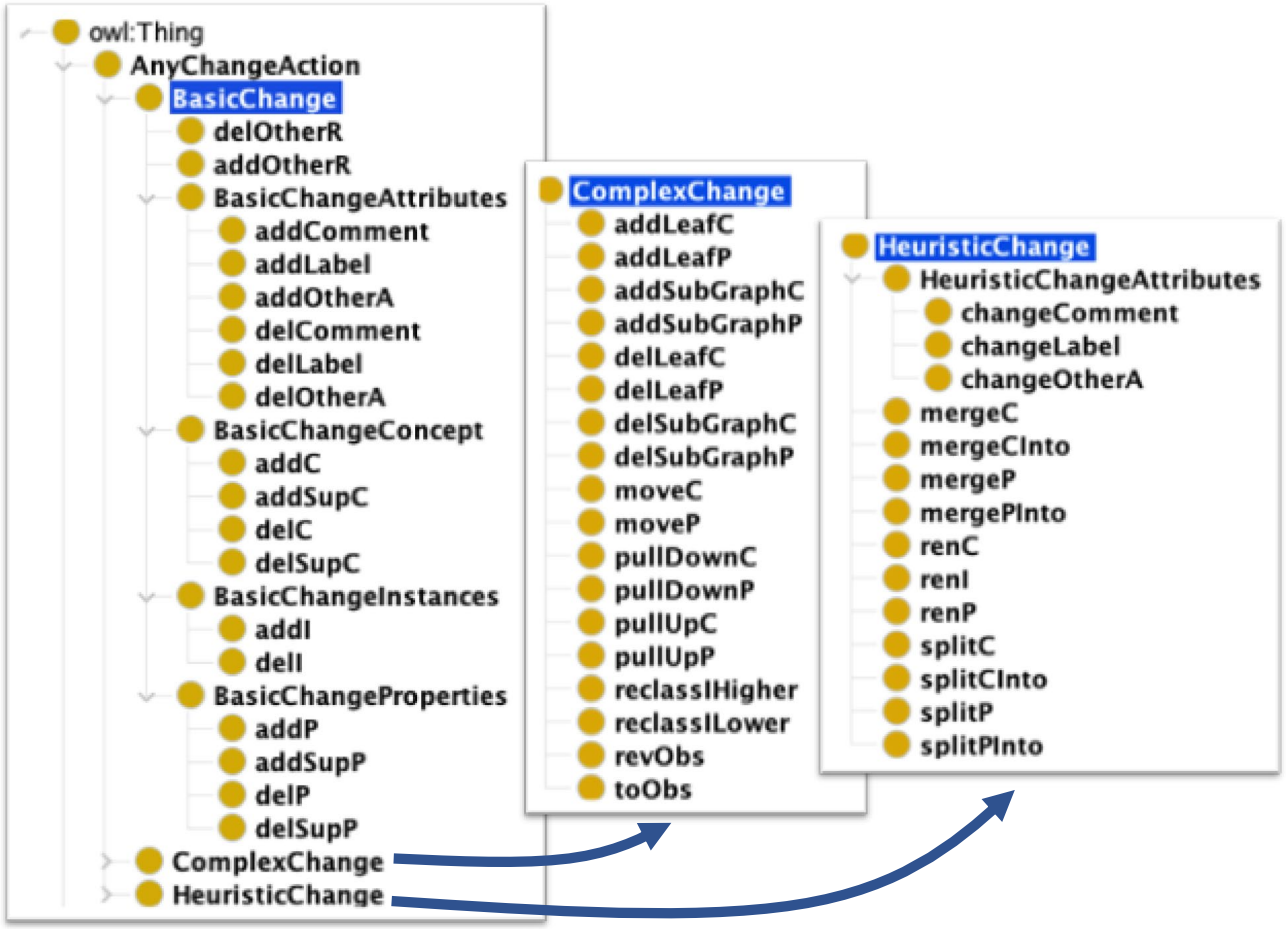
Illustration of the main classes of DynDiffOnto

The DynDiffOnto represents 60 different change actions (9 abstract classes and 51 leaf classes) as well as 9 object properties and 7 data properties. The definition of the classes started by analysing the changes produced by two different Diff methods (i.e. those presented in [12, 20]) when they were applied to 5 widely adopted ontologies in the medical domain. We compared them and mapped the changes with the same semantics in order to regroup them as classes of DynDiffOnto. Regarding changes that cannot be regrouped, we analysed them one-by-one and selected the classes according to the precision of the changes or their intuitiveness. The main inspiration comes from the work of Papavasileiou et al. [20]; we subsequently extended the definitions of some rules according to the work of [12], to finally add new rules from our own experience of evaluating the impact of changes in other artefacts presented in [8, 9]. By combining the idea of the class change attributes (from [12]) and the class change label and comments (from [20]), we created the BasicChangeAttributes and HeuristicChangeAttributes classes. This allows regrouping changes affecting comments, labels and other attributes that describe classes, individuals or properties. For instance, assuming that the following modifications were made in the old version of an ontology: adding the property rdfs:comment and assigning a textual description to comment each class. DynDiff will detect two different types of change actions: (1) the *addP* (add property) from the BasicChangeProperties; and (2) the *addComment* (add the text describing the class) from the BasicChangeAttribute. If one existing comment is slightly modified in a future version of the ontology, then DynDiff will detect a HeuristicChange (i.e. changeComment). These distinctions are important when we want to analyse the impact of changes in Semantic Mappings and Semantic Annotations because we use lexical and semantic similarity measures for this analysis [10]. The lexical similarity measures use information from attributes while semantic similarity measures take the relationships into account. We are currently working on a new representation format for the history of an ontology [28] that tracks changes in OtherA and OtherR classes of DynDiffOnto. Tables 1, 2 and 3 present the outcomes of our analysis including the label of the DynDiffOnto concepts and Fig. 1 illustrates the organization of DynDiffOnto.

**Table 1:** Basic change rules and their relations to [12] and [20] existing rules

Change actions	Description	Hartung [12]	Papavasileiou [20]
addC(c)	Add concept c	addC(c)	Add_Type_Class(a)
delC(c)	Delete concept c	delC(c)	Delete_Type_Class(a)
addP(p)	Add property p	-	Add_Type_Property(a)
delP(p)	Delete property p	-	Del_Type_Property(a)
addI(i)	Add instance i	-	Add_Type_Individual(a)
delI(i)	Delete instance i	-	Del_Type_Individual(a)
addSupC(r)	Add relationship r where the property = subclass	addR(r)	Add_Superclass(a,b)
delSupC(r)	Del relationship r where the property = subclass	DelR(r)	Del_Superclass(a,b)
addSupP(p,q)	Add subproperty p to property q	addR(r)	Add_Superproperty(a,b)
DelSupP(p,q)	Del subproperty p to property q	delR(r)	Del_Superproperty(a,b)
addComment(a)	Add attribute a where the prop. = comment	addA(a)	Add_Comment(a,b)
delComment(a)	Del attribute a where the property = comment	delA(a)	Delete_Comment(a,b)
addLabel(a)	Add attribute a where the property = label	addA(a)	Add_Label(a,b)
delLabel(a)	Del attribute a where the property = label	delA(a)	Delete_Label(a,b)
addOtherA(a)	Add attribute a where the prop. $\ne$ label or comment	addA(a)	Add_Property_Instance(a1,a2)
delOtherA(a)	Del attribute a where the prop. $\ne$ label or comment	delA(a)	Del_Property_Instance(a1,a2)
addOtherR(r)	Add rel r where the prop. $\ne$ subClass or subProperty	addR(r)	Add_Property_Instance(a1,a2)
delOtherR(r)	Del rel r where the prop. $\ne$ subClass or subProperty	delR(r)	Del_Property_Instance(a1,a2)

**Table 2:** Heuristic change rules and their relations to [12] and [20] existing rules

Change actions	Description	Hartung [12]	Papavasileiou [20]
renP($i_1,i_2$)	Rename property	-	Rename_Property($i_1,i_2$)
renI($i_1,i_2$)	Rename instance	-	Rename_Individual($i_1,i_2$)
mergeC($C_s,c_t$)	Merge multiple source concepts into one target concept	merge($C_s,c_t$)	Merge_Classes($C_s,c_t$)
mergeP($P_s,p_t$)	Merge multiple source properties into one target property	merge($P_s,p_t$)	Merge_Properties($P_s,p_t$)
mergeCInto($C_s,c_t$)	Merge multiple source concepts into one target concept $c_t \in C_s$	merge($C_s,c_t$)	Merge_Classes_Into_Existing($C_s,c_t$)
mergePInto($P_s,p_t$)	Merge multiple source properties into one target property $p_t \in P_s$	merge($P_s,p_t$)	Merge_Properties_Into_Existing($P_s,p_t$)
splitC($C_s,c_t$)	Split multiple source concepts into one target concept	split($C_s,c_t$)	Split_Classes($C_s,c_t$)
splitP($P_s,p_t$)	Split multiple source properties into one target property	split($P_s,p_t$)	Split_Properties($P_s,p_t$)
splitCInto($C_s,c_t$)	Split multiple source concepts into one target concept $c_t \in C_s$	split($C_s,c_t$)	Split_Classes_Into_Existing($C_s,c_t$)
splitPInto($P_s,p_t$)	Split multiple source properties into one target property $p_t \in P_s$	split($P_s,p_t$)	Split_Properties_Into_Existing($P_s,p_t$)
changeComment($c, att_{name},V_{Old},V_{New}$)	Change the value of the comment of c	chgAttValue($c, att_{name},V_{Old},V_{New}$)	Change_Comment(u,a,b)
changeLabel($c, att_{name},V_{Old},V_{New}$)	Change the value of the label of c	chgAttValue($c, att_{name},V_{Old},V_{New}$)	Change_Label(u,a,b)
changeOtherA($c, att_{name},V_{Old},V_{New}$)	Change the value of the attribute of c	chgAttValue($c, att_{name},V_{Old},V_{New}$)	-

**Table 3:** Complex change rules and their relations to [12] and [20] existing rules

Change actions	Description	Hartung [12]	Papavasileiou [20]
pullUpC($c,B_1,B_2$)	Move c to a higher position in the hierarchy	-	Pull_up_Class($c,B_1,B_2$)
pullUpP($p,B_1,B_2$)	Move p to a higher position in the hierarchy	-	Pull_up_Property($p,B_1,B_2$)
pullDownC($c,B_1,B_2$)	Move c to a lower position in the hierarchy	-	Pull_down_Class($c,B_1,B_2$)
pullDownP($c,B_1,B_2$)	Move p to a lower position in the hierarchy	-	Pull_down_Property($p,B_1,B_2$)
moveC($c,B_1,B_2$)	Move c horizontaly	move($c,B_1,B_2$)	Move_Class($c,B_1,B_2$)
moveP($p,B_1,B_2$)	Move p horizontaly	-	Move_Property($c,B_1,B_2$)
recastI($i,B_1,B_2$)	Change type of i to $B_2$	-	Reclassify_Individual($i,B_1,B_2$)
toObsC(c)	c becomes obsolete	toObsolete(c)	-
revObsC(c)	revoke obsolete status of c	revokeObsolete(c)	-
toObsP(p)	p becomes obsolete	toObsolete(p)	-
revObsP(p)	revoke obsolete status of p	revokeObsolete(p)	-
addLeafC(c,p)	add c as leaf of p	addLeaf(c,p)	-
delLeafC(c,p)	del c as leaf of p	delLeaf(c,p)	-
addLeafP($p_1,p_2$)	add leaf property $p_1$ below class $p_2$	-	-
delLeafP($p_1,p_2$)	del leaf property $p_1$ below class $p_2$	-	-
addSubGraphC(c,B)	add a subgraph with root c and children B $p_1$ below class $p_2$	addSubGraph(c,B)	-
delSubGraphC(c,B)	del a subgraph with root c and children B $p_1$ below class $p_2$	delSubGraph(c,B)	-
addSubGraphP(p,B)	add a subgraph with root p and children B $p_1$ below class $p_2$	-	-
delSubGraphP(p,B)	del a subgraph with root p and children B $p_1$ below class $p_2$	-	-
renC($i_1,i_2$)	rename C from $id_1$ to $id_2$	-	Rename_Class($i_1,i_2$)
reclassIHigher($i_1,i_2$)	When the instance is reclassified to a more generic class (i.e., the new class is an acester of the old class)	-	Reclassify_Individual_Higher($i,B_1,B_2$)
reclassILower($i_1,i_2$)	When the instance is reclassified to a more specific class (i.e., the new class is subsumed by the old class)	-	Reclassify_Individual_Lower($i,B_1,B_2$)

In order to favour reusability, we followed the FAIR principles and used dedicated methods [29] and tools to evaluate and improve the FAIRness of DynDiffOnto. Each new concept is described with a definition. We also attached standard metadata (e.g. those provided by the Dublin Core) to the ontology to better describe its content as advocated by the FAIR principles. DynDiffOnto is publicly available at the https://git.list.lu/dynaccurate/change-ontology permanent URL. We have associated this resource with a creative commons licence to clarify the reuse of the ontology. Moreover, the documentation of the ontology was generated using the WIDOCO software application [30]. Finally, we used the Oops! application [31] to detect potential inconsistencies of DynDiffOnto and then corrected them.

### DynDiff for comparing large and dynamic ontologies

In this section, we explain how DynDiff computes the changes between two ontology versions. The granularity of the description of the changes is an important aspect that provides rich information about the evolution of the ontology. The change description can be low-level, when composed of add and remove changes only, or high-level, when low-level changes are regrouped in order to report a more intuitive interpretation of these changes. As Fig. 1 shows, the change actions were regrouped into three different categories: Basic, Complex and Heuristic changes.

- Basic changes: this is a set of low-level changes that are meant to be machine-understandable. Examples would be the addition of a Concept (abbreviated as addC). These are obtained by identifying triples that exist in a recent version but not in the older one.
- Heuristic changes: These are composed of at least one basic change and the main characteristic of these high-level changes is that they depend on the mappings computed through the user-selected matcher (i.e. the method chosen by the user to map ontological entities before and after their evolution). Since this method is based on heuristics, we named this group heuristic changes. An example would be the split of a concept (abbreviated as splitC ). This type of change requires the determination of a mapping between the original concept (older version) and all other concepts from the new ontology that compose the split. This changes significantly depending on the matcher used and thus is non-deterministic. This type of change is more easily understood by humans. Indeed, it is easier to understand a split rather than a combination of basic changes. Thus, the more complex changes can be detected, the more the evolution of the ontology will be understandable for humans. Moreover, from a system point of view, having higher-level inferred-diff-annotations facilitate the maintenance of ontology mappings or semantic annotations as demonstrated in [9, 28].
- Complex changes: in contrast to the previous category, these high-level changes are deterministic, independent of the matcher, and use well-defined logic rules to combine basic changes. This is why we separated them from the heuristic changes, as it would make the determinism more transparent to the user. An example is addSubGraphC, which regroups all concepts and relationships from a new branch of the ontology.

Algorithm 1 shows the sequence of instructions performed. In this work, we implemented the rules proposed by [20] to generate the low-level triples, basic changes and complex changes. We adopted the approach of [12] to compute the heuristic changes. This decision ensures that all proofs presented in [20] also apply to this work. Readers can find a detailed explanation of the algorithm in the referenced articles.

The set of low-level changes, including all added and removed triples, is computed following the algorithm described in [20]. The triples are used as the input for the matcher (algorithm 2, line 3) and are also used to compute the *Basic changes* (algorithm 2, line 4). Examples of basic changes generated from the triples are addC, delA, addI, etc. By combining the basic changes, the heuristic and complex changes can be identified. Thus, the elements of the set of basic changes that are used/consumed to create more complex changes are deleted from the basic changes set (algorithm 3, line 9). This deletion action follows the principle of unambiguity (avoiding overlapped changes) proposed by [20]. The matcher searches for potential links between the triples and expresses these links as mappings. Several matching systems aggregate different categories of matcher. This can be done in either a sequential manner, where the output of one is fed to the next matcher; or in a parallel composition, where the algorithms run independently of each other and their results are later combined given certain criteria. Once these computations have concluded, a global strategy has to be executed to get the final optimized mappings. These strategies include trimming, which applies thresholds to keep the most relevant results, e.g. the maximal weight similarity [32]. Moreover, recent approaches have begun to include additional post-computing processes like checking to prune illogical mappings or even full-fledged repair tasks to ensure the coherence of the final results [33]. In [34], readers can find different examples of these categories and better understand how to use them. Given the amount of matcher available, we narrowed our search/evaluation to those with a good performance in the Ontology Alignment Evaluation Initiative (OAEI)^3^.

For this work, we present the results obtained when adopting the same matcher as used in COnto-Diff. We had to add a small modification in the matching rules in order to allow mappings to be generated for properties and instances. Note that the difference in the mapping rule is:

COnto-Diff rule: $r \in R(Relationships) \wedge a,r \in O_{old} \wedge b \in O_{new} \wedge r_{source} = a \wedge r_{target} = b \wedge r \in$ $\{``part\_of'',``is\_a'',``synonym''\} \rightarrow {\textbf {match(a,b)}}$

DynDiff rule: $a \in O_{\text{ old } } \wedge b \in O_{\text{ new } } \wedge match C(a, b) \wedge a \ne b$ $\wedge \lnot isObsolete(a) \wedge \lnot isObsolete(b) \rightarrow$ create $[{\textbf {mapC(a, b)}}]$

Similar DynDiff rules for properties and instances were defined. Since COntoDiff uses the OBO format to compare ontologies, the restriction on the relationships is justified. DynDiff uses subsumption relationships defined by RDFS (e.g. rdfs:subClassOf) and several formats for synonyms (e.g. skos:altLabel). The reasons for adopting the same matcher are: (1) To compare the performance of both approaches fairly; (2) To demonstrate the efficiency of our approach to identify changes.

Based on the mappings and the basic changes, the algorithm will compute probabilistic relations between the basic changes in order to represent more abstract changes such as split, merge, change label, etc. (algorithm 3, line 6). The Complex changes consume the basic changes according to a set of deterministic rules in order to represent more abstract change actions (e.g. moveC consumes addSupC and delSupC) (algorithm 3, line 8). The set of all rules used in this project can be downloaded from the project repository^4^.

The algorithms below will show how these rules were used to implement DynDiff. Algorithm 1 presents the *Compute Diff* change detection algorithm:

**Figure:**
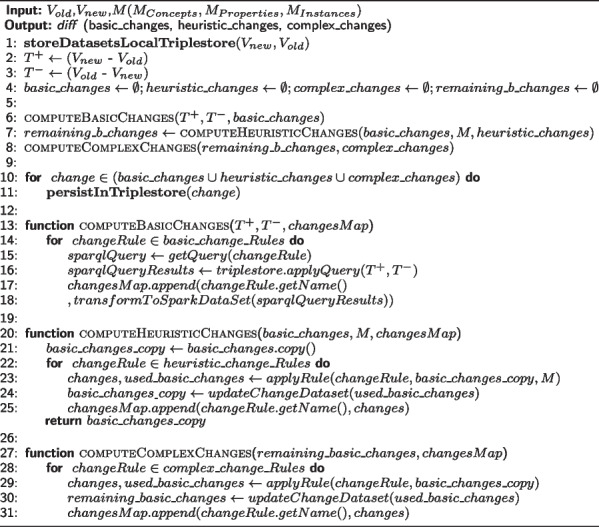
**Algorithm 1** Compute diff change detection algorithm:

**Figure:**
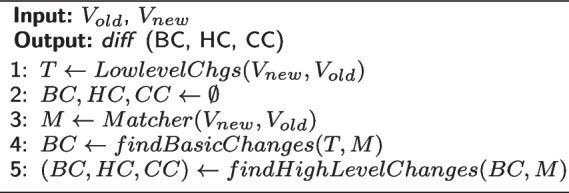
**Algorithm 2** Compute diff:

**Figure:**
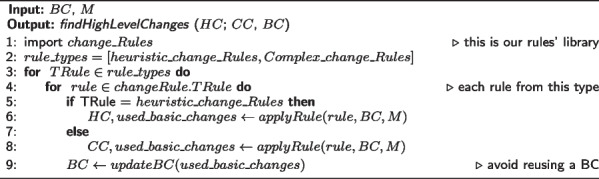
**Algorithm 3** Finding High-Level Changes

As explained in [20], in theorems 7.2 and 7.3, the complexity of algorithm 1 is $O(N^2)$ where N is the size of the input: ontology versions $V_{old}$,$V_{new}$, and mappings which are given by a chosen matcher $M(M_{Concepts},M_{Properties},M_{Instances})$. This complexity can be explained by the size of the input of the *PullUpConcept*(*concepta*, *oldParents*, *newParents*) complex changes where the size of (*oldParents*, *newParents*) is equivalent to the size of the ontology.

### Implementation

**Fig. 2:**
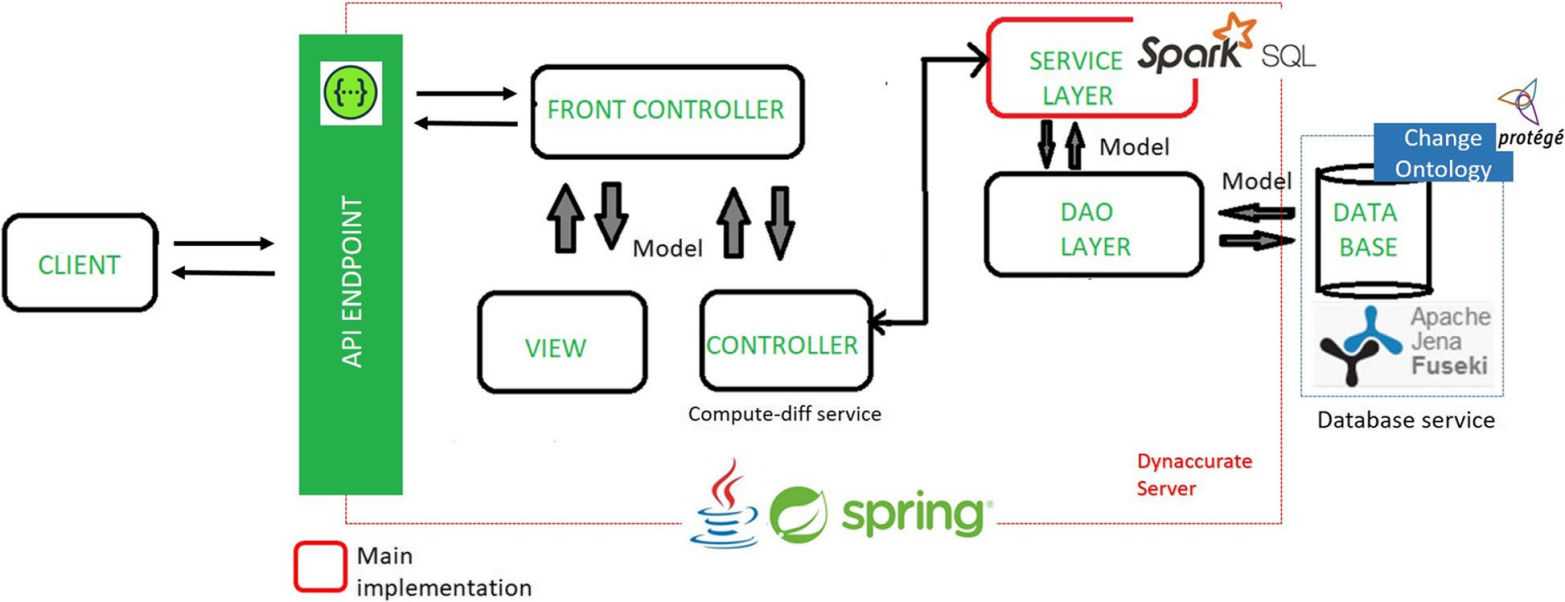
*DynDiff* algorithm service layout with its technology dependencies

The application functions under the MVC (model - view - controller) design pattern and the implemented service is exposed as a RESTful API. Figure 2 shows the overall layout of the service architecture and its associated technologies:

The client makes requests to the API through the API endpoint. It goes to the Front Controller of the application that serves as a handler and dispatches the respective service controller, as shown in Fig. 2. Then, it calls the service layer, where the implementation of the abovementioned algorithms are executed. The service layer loads the data stored in the triplestore^5^ through the data access object (DAO) layer into an in-memory Spark^6^. The intermediary Spark data layer is accessed when we need large-scale data processing capabilities, e.g. diff between 400.000 concepts and the Triplestore when we need to compute, logical queries, e.g. subClassOf. The final Diff is stored in the triplestore database. This architecture decouples the algorithms and the databases, allowing triplestores to be switched easily, if necessary.

All the application’s API architecture is developed using the Spring Framework and Java 11. The API is documented using Swagger, which follows the OpenAPI Specification version 3.0.3 which defines a standard, language-agnostic interface to RESTful APIs and allows humans and computers to discover and understand the capabilities of the service without access to source code, other documentation, or through network traffic inspection. Spark SQL is used to manipulate the data for the services, before storing it in Apache Jena’s triplestore, which is accessed by the Fuseki Sparql endpoint. The triplestore’s schema is the proposed change ontology. The service is deployed with the help of Apache Maven and as a Docker container which is orchestrated with the other services using Kubernetes.

## Results

DynDiff has been experimentally evaluated on large ontologies in the biomedical domain. Our goal was to assess the performance of the algorithm on existing tools in terms of execution time, as well as its ability to identify the right change actions. We also propose additional experiments to evaluate the influence of the ontology matcher on our method.

### Materials

During this evaluation, we used seventy-three versions from seven different ontologies. Table 4 provides the average number of classes (C), properties (P), individuals (I), subsumption relationships (R) and attributes (A) that are contained in the various version of these ontologies.

**Table 4:** Average number of concepts ($\textbf{C}$), properties ($\textbf{P})$, individuals ($\textbf{I}$), subsumption relationships ($\textbf{R}$) and attributes ($\textbf{A}$) in the different versions of each of the datasets. The *Versions* column shows the versions collected

Acronym	Versions (#)	Average number of				
		$\textbf{C}$	$\textbf{P}$	$\textbf{I}$	$\textbf{R}$	$\textbf{A}$
ICD9CM	2005AA - 2016AA (12)	21,765	2	0	21,765	54,572
MESH	2005AA - 2016AA (12)	25,820	2	0	34,899	215,971
NCIT	2005AA - 2016AA (12)	70,396	2	0	79,525	179,983
SNOMEDCT	2005AA - 2016AA (12)	309,572	2	0	496,084	800,158
GO	01072017 - 21072020 (4)	52,574	54	0	492,651	395,285
IOBC	1.0.0 - 1.4.0 (7)	115,114	84	59,346	707,970	904,443
CIDO	01262020 - 06142020 (14)	3,599	165	294	15,368	25,407

We collected these ontologies from BioPortal [5]. For each of the dataset versions, the initial and last ID used are listed in the “versions” column, as well as the number of versions (in parentheses). We used the notation AA to indicate that for these ontologies we selected the latest version published in January of each year. For Gene Ontology (GO), Interlinking Ontology for Biological Concepts (IOBC) and the Ontology of Coronavirus Infectious Disease (CIDO) we indicate in the table the first and last version used.

### Experimental method and metrics

The main research objective of this work deals with the scalability, robustness and completeness of the method. The scalability and robustness aspects deal with the ability of the approach to compare large ontologies while the completeness aspect deals with the ability of the approach to identify a larger set of ontological changes compared with state-of-the-art methods. To evaluate these objectives we executed DynDiff and COnto-Diff algorithms in the same computing environment and used the following metrics to analyse the results obtained.

- Execution time: With this metrics we will be able to compare the computation time required by DynDiff and COnto-Diff to measure the scalability and robustness of both algorithms. Here we studied two main indicators:
  - Composition: we studied the composition of the execution discerning between the loading and actual algorithm execution time. We did this to understand the performance of the DAO (loading) and service (algorithm execution) layers. Additionally, it provides a benchmark of the algorithms based on the Apache Jena Fuseki implementation.
  - Trends and behaviours: to understand the behaviour and trends of the execution time given a set of characteristics (like input size, output size and result composition in terms of changes, among others); in addition, we analysed the outcomes to evaluate the scalability of the algorithm in terms of these characteristics.
- Composite to low-level ratio: for this indicator, the low-level are the basic changes while the composite changes are the higher level changes, regrouping the complex and heuristic ones. We look for the ratio between composites and low-level changes to analyse how good the algorithm to compute them is in terms of completeness. Note that composite changes are harder to compute and require more computational time/resources.
- % change tool comparison: The following indicator is used to compare the results of DynDiff (DD) with those of COnto-Diff (CO) [12]:
  2 $$\begin{aligned} \%ChangeTools_{type}= \frac{AVG(N_{DD,type}-N_{CO,type})}{AVG(N_{CO,type})}, \quad type \in \{Basic, Composite\} \end{aligned}$$
  where N is the number of changes of a given tool and of a given type: basic or composite (complex or heuristic). The COnto-Diff algorithm serves as a ground truth since it was also compared (in previous work) with other existing solutions like PromptDiff. We compared the “execution time” indicator and evaluated the proposed “%changeTools” indicator. This analysis also enabled us to explain the differences in the composite-to-low-level ratio. The methodology here is the same, except that there is no API service (and thus no UI); everything is set up through the IDE and the intermediary and final results are kept in a local database.
- % change matcher comparison : To compare with our default matcher, we selected the AML (Agreement Maker Light) matcher^7^ because it is an open source (we were able set up a comparable environment and execute the code) and because of the good performance of this matcher in the OAEI from 2019. By changing the default matcher of our tool, we are able to see the effect of the new matcher on the performance of our algorithms. We used the following equation to measure the matcher impact:
  3 $$\begin{aligned} \%ChangeMatchers = \frac{AVG(N_{AML}-N_{DM})}{AVG(N_{PA})} \end{aligned}$$
  where DM refers to the default matcher and AML to the new one. N refers to the amount of heuristic changes for the given matchers (DM, AML). The methodology to gather the results of the new matcher is the same as that described for the default one, although we limit the analysis to three ontologies: SNOMEDCT in order to evaluate the results in a large ontology; ICD9CM because it is a medium-sized ontology; and CIDO as it also contains properties and instances.

### Evaluation of DynDiff

The first set of experiments examines the relationship between the execution time (in seconds) of DynDiff and the total amount of detected changes (referenced as “composition” in the previous section).

**Fig. 3:**
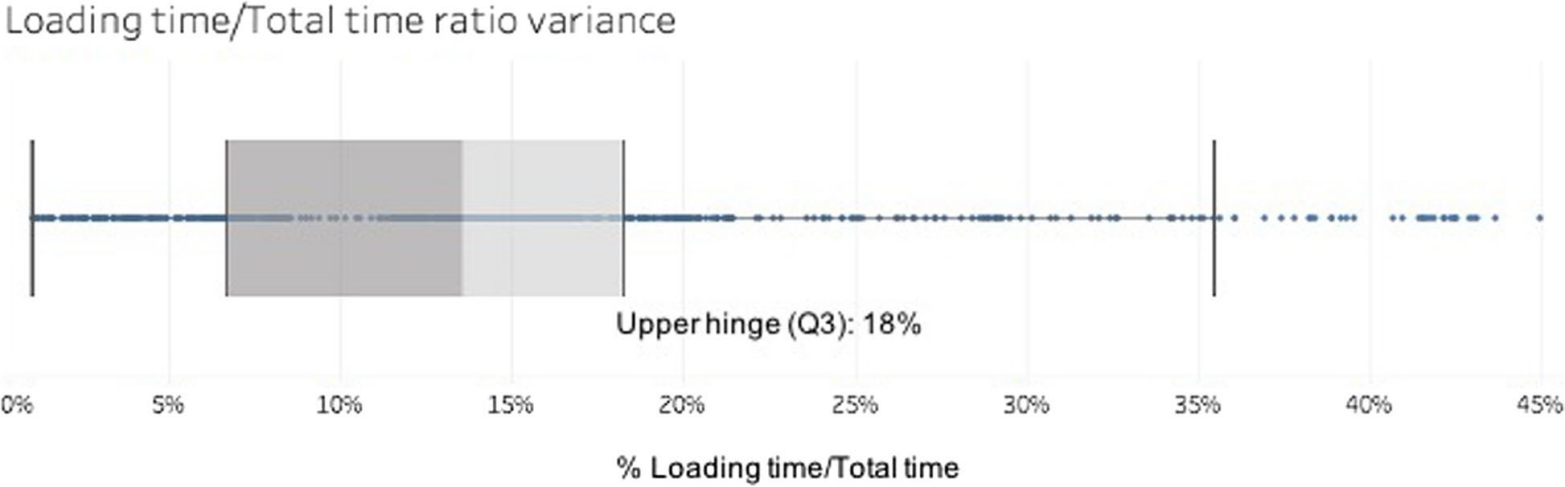
Variance of the ratio of loading time to execution time through all iterations of the proposed ontologies

The total execution time is made up of two processes: the loading time of the local triplestore and the actual execution time of the algorithm. As shown in Fig. 3, in at least 75% of the cases, the loading time represents less than 18% of the total execution time (see the upper hinge). The maximum loading time was observed for GO ontology and represents 34% of the total execution time, the minimum loading time was observed for cido ontology and represent is 4% of the total execution time. The high loading time is probably due to the parsing step undertaken to transform the original OBO format of GO into OWL. The other ontologies have a native OWL format, avoiding the parsing step. We observed that the parsing process adds extra triples to GO, such as blank nodes, that require more processing time to load and to recompose the graph in memory.

**Fig. 4:**
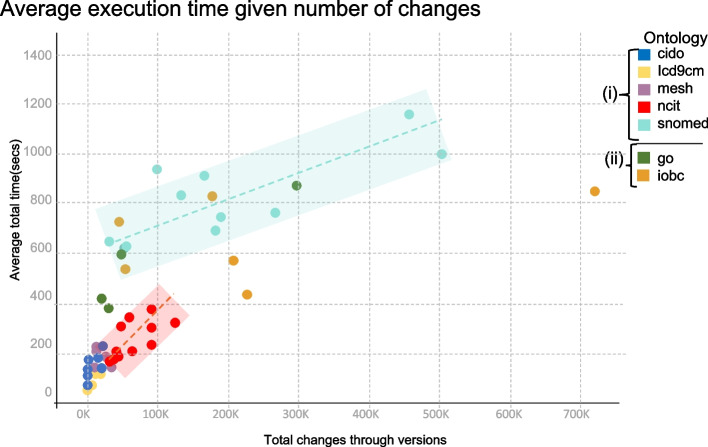
Average execution time (secs) vs total of changes per computed diff between consecutive versions of ontologies

The next step of our evaluation will better detail how the execution time of the algorithm is used. For this, we will start with the analysis of “trends and behaviours”. Figure 4 shows the total amount of changes computed per ontology (diff between consecutive versions) versus the execution time. The plotted execution time represents the average of 10 iterations for each Diff calculated between two consecutive versions. The goal of this analysis is to determine if the execution time increases proportionally to the number of changes in order to validate the scalability of our algorithm.

As shown in Fig. 4 the execution time follows two patterns: (i) reference, (ii) high variance. The reference pattern is characterized by the fact that, for each ontology, there is a proportional relation between the execution time and the number of changes. Notice that the ratio can be different for different ontologies (as indicate by the red, for NCIt, and green, for SNOMED CT, doted lines in Fig. 4). According to our findings, two ontologies do not follow the reference pattern: GO and IOBC. For instance, GO (with many concepts and few instances) has an execution time varying between 300s and 850s to compute 30K to 300K changes. IOBC (with few concepts and many instances) expends between 400s and 900s to compute 50K to 700K changes. For both ontologies, we observed a non-linear increase of the execution time vs number of changes. In the next steps of our analysis, we will evaluate the potential impact of other factors on the execution time of our algorithm.

**Fig. 5:**
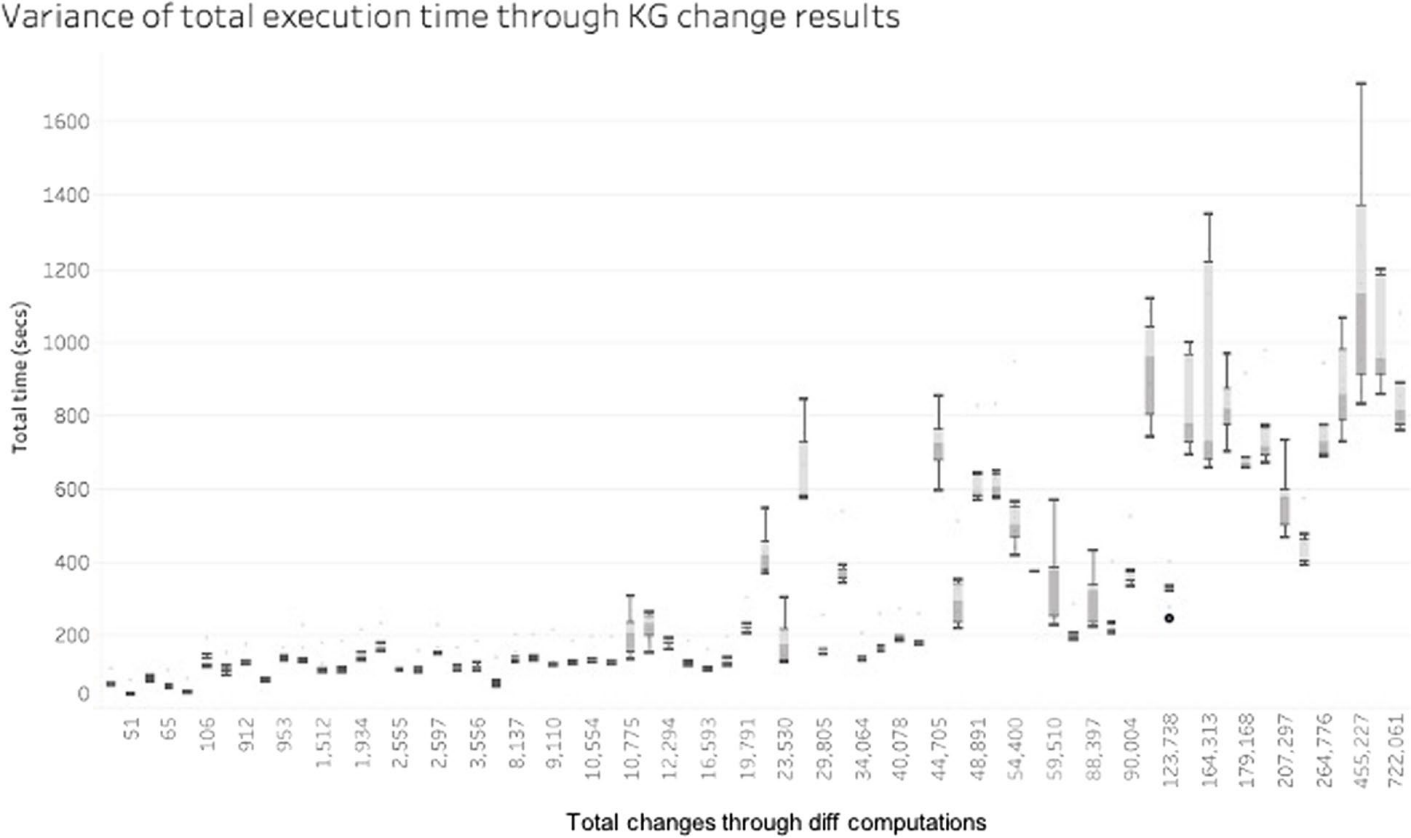
Average of execution time (secs) vs Average of total changes for DynDiff

The first analysed factor was the computation environment. Figure 5 shows that there is an inherent variability among the 10 iterations to compute the diff between the different versions. Here we see the total amount of changes produced, and a box-plot quartile analysis over the 10 iterations done. This is mainly due to the start-up time used by the Java Virtual Machine in the first iterations as well as the randomness intrinsic to not using a dedicated server or machine for the computation.

Additionally, Spark is used here for resource management and is known to have high costs at the start and finish of jobs according to the benchmark done by [35].

The next series of experiments will demonstrate that computing composite changes can have an impact on the execution time. This is an important analysis because our approach proposes to compute more composite changes than the other existing approaches.

### Composite vs low-level changes

**Fig. 6:**
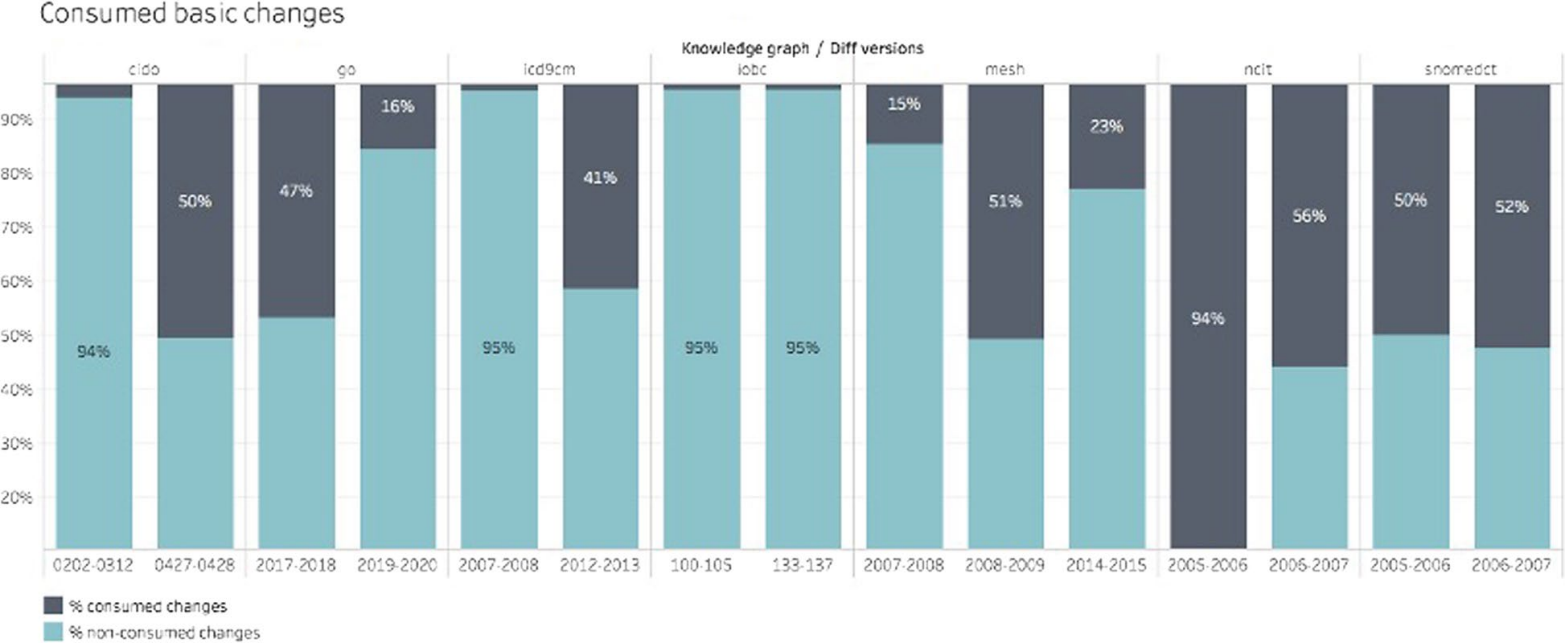
Percentage of consumed basic changes by computed diff. We present some examples computed for seven different ontologies

One of the main objectives of this work is to identify and characterize changes that are not only machine- but also human-interpretable. For this reason, it is important to evaluate the capacity of DynDiff to transform the basic changes into composite ones. Figure 6 shows the percentage of basic changes consumed by the composite changes (consumed basic changes/total basic changes) for 2-3 different computed diffs per ontology.

Note that IOBC consumes few basic changes. The most frequent change observed in this ontology was the deletion of labels, which is classified as a basic change. It can partially explain the low execution time required for 700K changes, as shown in Fig. 4. In contrast, the algortithm consumes (on average) more than 50% of the basic changes for NCIt and SNOMEDCT. For example, for NCIt 2005-2006 diff computation, the algorithm was able to identify a large subgraph that was composed of a little more than 48k concepts (addSubgraphC), consuming almost all the basic changes. The consequence of this higher quantity of basic change consumption is observed in Fig. 4 by a higher execution time for these ontologies. Thus, the reduction of the execution time can be obtained by observing the evolution of an ontology and selecting the composite changes that are useful for the end-users. The analysis of the composite changes is closely related to the context and the use that will be made of this information. For instance, we plan to use the types of changes to evaluate the impact of these changes on the mappings [9] and on the semantic annotations of documents [10]. The ultimate goal is to automatically update the mappings and annotations according to the evolution of the ontologies. For this context, the set of rules that we selected covers all our needs. Other contexts would require only a sub-set of these rules, resulting in a reduction of the calculation of composite changes and consequently in a reduction of the execution time for some ontologies. However, the bottlenecks can also come from the matcher and the rules for detecting heuristic changes. According to the chosen matcher and the type of ontology that it will be applied for, the performance of our algorithm can be different. This will be detailed later when the differences in the results given by the matchers are analysed.

**Fig. 7:**
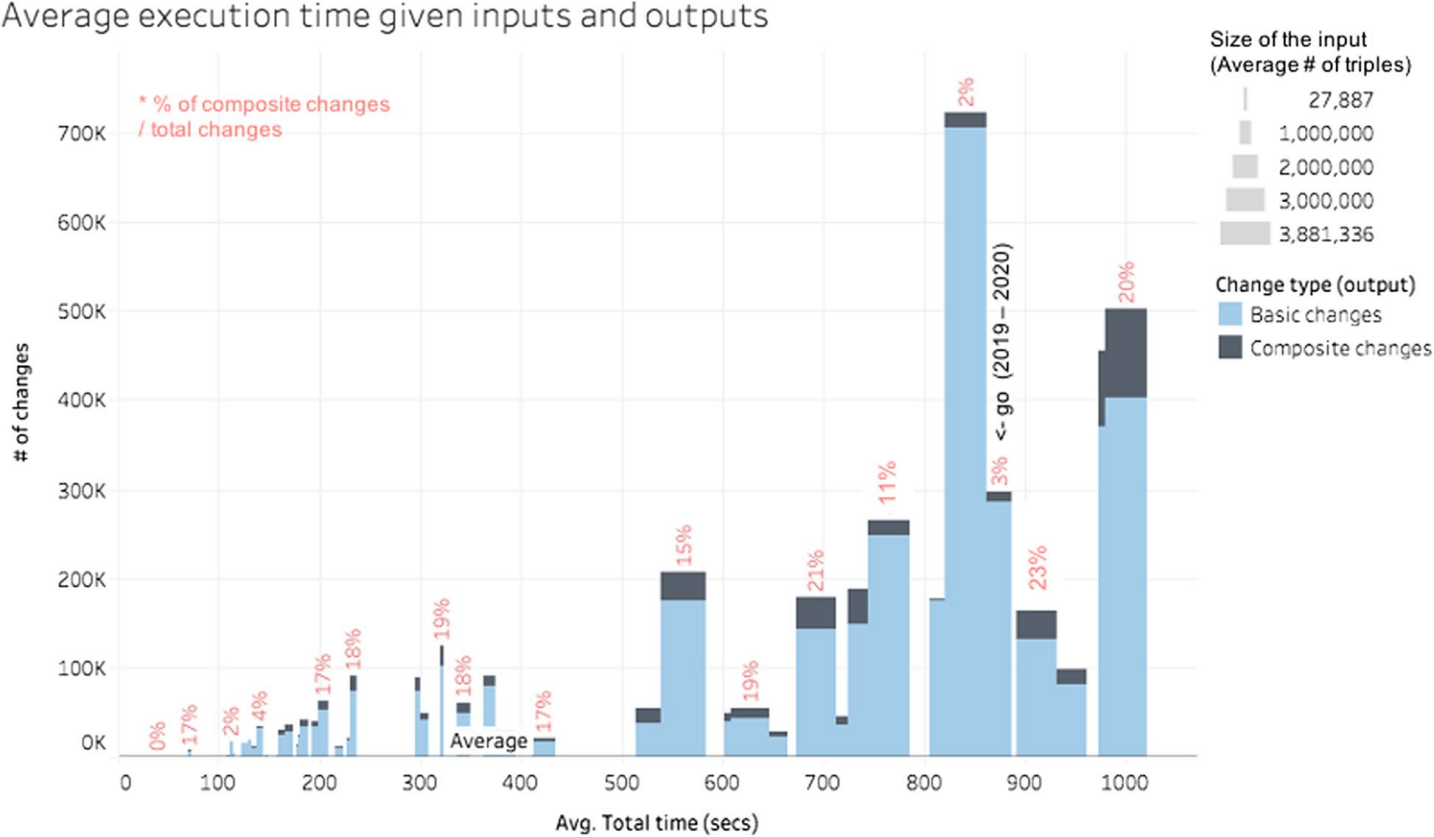
Effect of number of changes (output), input size of version ontologies (width), and the percentage of complex changes in average execution time (secs)

Combining the size of the ontology, the quantity of detected changes, and the ratio of composite changes enables a multi-perspective view of the execution time, as shown in Fig. 7. The width of the bars represents the size of the input versions (number of triples representing concepts, relationships, attributes, properties and instances) and the height of the bars represents the number of detected changes. The colours inside the bars distinguish basic changes from composite ones. The ratio between basic and composite changes is also indicated by a red label. The horizontal axis of the figure indicates the average execution time of the algorithm. Note that increasing the input (width) or output (height) cannot justify per se the increase of the computation time. However, if the quantity of composite changes is also taken into account, the correlation becomes clearer. The bar furthest to the right (with the higher execution time) has the following characteristics: large input ontologies and high number of detected changes, with 20% of them being composite changes. Although this does not explain all the cases, it is definitively clear that all execution time over 500s has a large ontology as its input even if the number of the detected changes is not that big. For instance, one diff computed for SNOMEDCT has an execution time greater than 900s and the number of changes is close to 100K. Thus, the type of ontology also impacts the execution time. By comparing Figs. 7 and 4, we can observe that it is time-consuming to compute diffs for SNOMEDCT. One hypothesis is that the sophisticated hierarchical relationship within SNOMEDCT could partially justify this high execution time. Deeper analysis is necessary to demonstrate this hypothesis. But, this demonstration is out of the scope of our work since it impacts all Diff algorithms in the same way.

The next step will be to compare our approach with the state-of-the-art in this domain, i.e. COnto-Diff.

### Comparison with COnto-Diff

The close collaboration with the University of Leipzig and in particular, with the researchers developing COnto-Diff tool [12] was crucial to the setup of an execution environment in order to fairly compare the tools. The rules for Properties $\textbf{P}$ and Instances $\textbf{I}$, cannot be compared with COnto-Diff because COnto-Diff was not designed to analyse these elements. Inspired by the work of [20], our approach added this more granular view to characterize the changes with, for instance, addSubClass, addSubProperty, addOtherRelationship, etc.

### Execution time

**Fig. 8:**
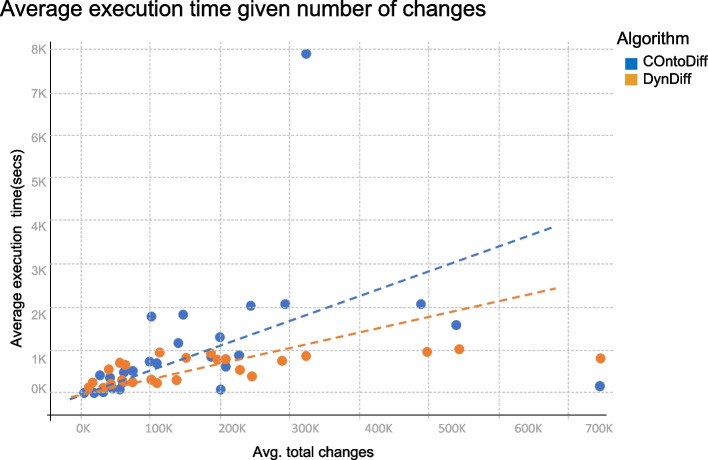
Comparison of average execution time (secs) vs total changes per computation between consecutive versions of proposed ontologies

The first comparison’s factor is the total execution time spent to compute the Diff. In Fig. 8 we can observe the total changes computed by each algorithm (COnto-Diff and DynDiff) for the consecutive versions of the seven ontologies proposed (we used the average execution time of its 10 iterations). Note that on average, the execution time of DynDiff varies less than that of COnto-Diff, especially for larger ontologies or for a large number of changes. The trend of execution time of both algorithms are presented as dotted lines in the figure. For instance, when there are more than 230K changes, COnto-Diff needs twice as much time as DynDiff to compute the changes. The IOBC exception (700K changes) is observed because COnto-Diff does not compute changes in instances (what is mostly the case for IOBC). Moreover, the GO 2019-2020 was excluded from the analysis because COnto-Diff was unable to compute the diff.

**Fig. 9:**
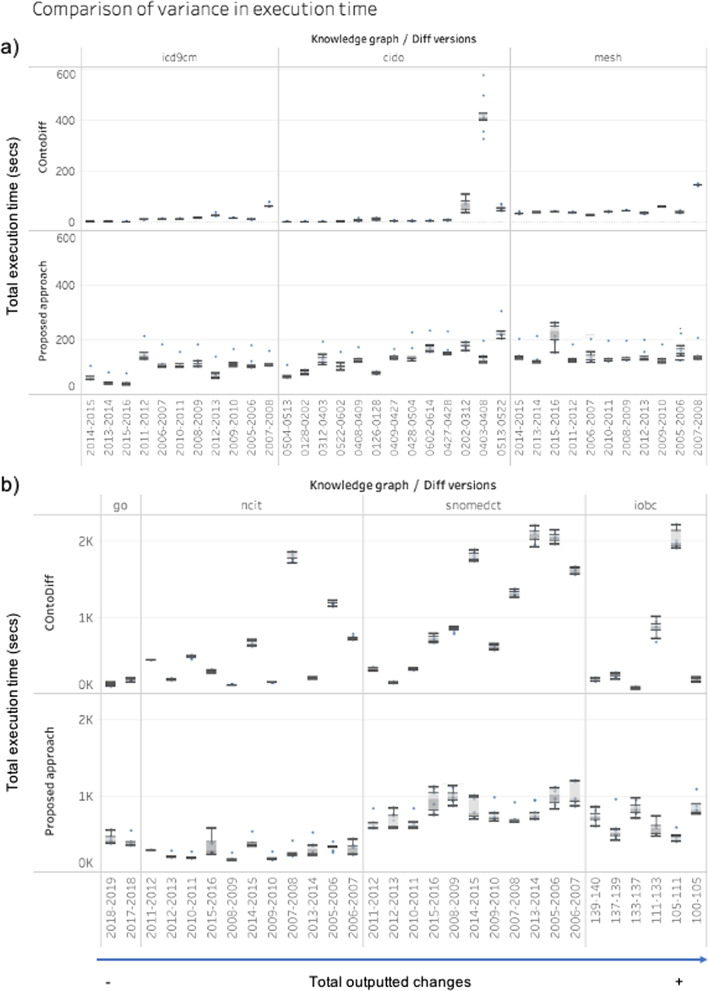
Comparison of the execution time per iteration and its variance for each of the computed diffs. **a** small to medium and **b** large sized ontologies

The variance in the total execution time for each approach can be seen in Fig. 9. Note the good performance of COnto-Diff for smaller ontologies. This can be explained by the bigger quantity of rules to test and the higher loading time for DynDiff. The novelty is that DynDiff performs well for larger ontologies and even better for larger ontologies with a big quantity of changes which confirms the robustness of DynDiff. The box-plot presents the total execution time per Diff. We regrouped the Diffs by ontology and, within each group, the values are ordered (ascendant) by the number of computed changes.

By looking more closely at the variability in the execution time, COnto-Diff shows a higher variance for CIDO on some diffs, such as 0403-0408 and 0202-0312, which has the highest amount of complex changes in this group. These two diffs contain up to 300% more complex changes than the other versions. For larger ontologies, the variability is more evident, such as for IOBC 105-111. This variability can be caused by certain characteristics of the ontology. For instance, IOBC 105-111 uses labels in both Japanese and English. This high variability was not observed with DynDiff. However, for MESH 2015-2016 or NCIt 2015-2016, we observe a higher variability with DynDiff. Since the quantity of changes in these two ontologies are quite small (near 200), we suspect that the start-up and finishing overheads given by Spark had an impact on our experiments.

**Fig. 10:**
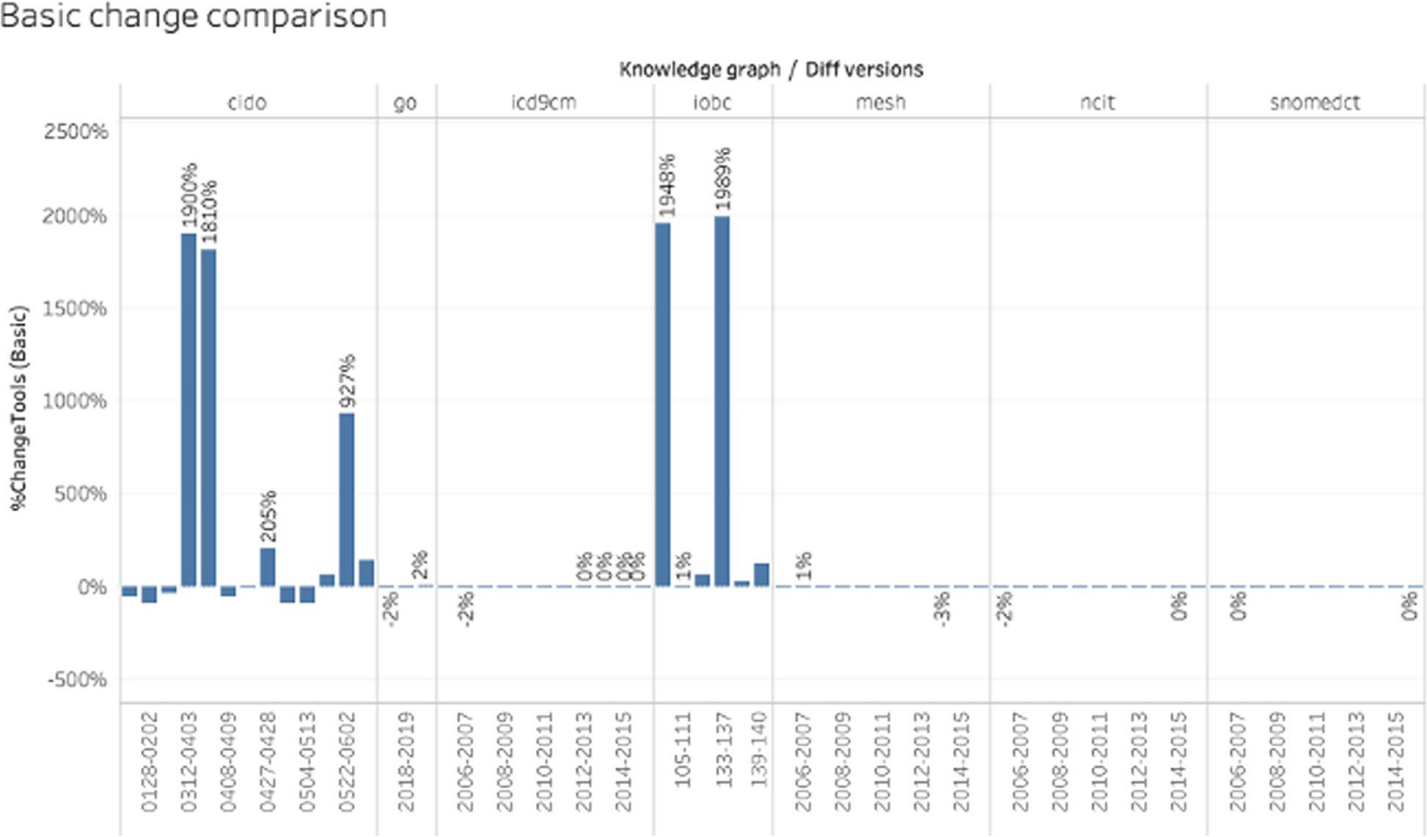
Comparison of the basic changes computed by DynDiff vs ones computed by COnto-Diff

As an indicator of accuracy, we compare the changes detected by DynDiff with those from COnto-Diff. We defined the $\%changeTools_{type}$ metric (see Eq. 2) to quantify the differences of changes computed by each tool. We start by comparing the basic changes as shown in Fig. 10. Having a value close to 0% indicates that the same number of changes were found by both tools. The differences observed, such as for IOBC and CIDO, express the capacity of either DynDiff or COnto-Diff to characterize more types of changes than the other one. If the value is positive, then DynDiff detects more changes, if it is negative, then COnto-Diff detects more changes. For IOBC and CIDO, a plausible explanation for the differences is based on the inability of COnto-Diff to process the labels in Japanese as well as on the inability of COnto-Diff to identify the changes in instances and properties (since it does not have rules for that). Similar results are also observed for the analysis of composite changes. The slight differences of 2-4% for the ontologies are mainly due to the rules for heuristic changes.

The negative percentages are due to the ability of COnto-Diff to compute *addInnerC* and *delInnerC* in order to detect the concepts added or deleted between a leaf and non-leaf node. Our approach uses *addSubGraph* and *delSubGraph*, which group several add/delete operations into a more complex operation. This decision generates more changes for COnto-Diff, expressed as negative values in the figure. This analysis shows that both approaches behave similarly for ontologies that do not have changes in properties or individuals, but only DynDiff can identify these kinds of changes making DynDiff more complete in its ability to detect high-level changes.

### Influence of the matcher

The reason for evaluating several matchers is to show their potential impact on the results and to highlight the importance of choosing one matcher wisely. The comparison will be between the built-in matcher and the AML^8^ matcher version 3.1. Before selecting AML, we evaluated 7 matchers (all matchers that performed well for large biomedical ontologies in the OAEI 2019 [36]). In this evaluation, AML had the highest overall F-measure and took fourth position in terms of execution time. From a technical perspective, our experiments were executed on an Ubuntu 18 Laptop with an Intel Core i5-6300HQ CPU @ 2.30GHz x 4 and 15Gb of RAM. The ontologies used were SNOMEDCT (with 306.500 concepts on average) and NCIT (with 66,724 concepts on average).

**Fig. 11:**
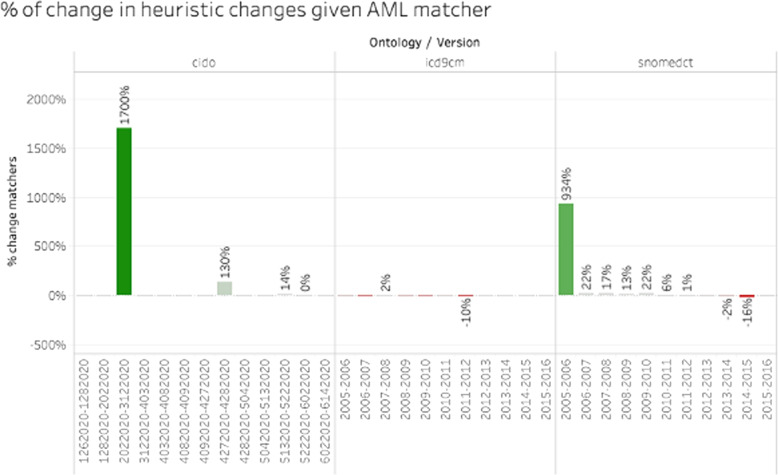
Comparison of increase in heuristic changes using AML matcher

For the experiments presented in Fig. 11, we decided to deactivate the feature word-matcher from AML because when applied to our dataset, it generates an “outof-memory” error for large ontologies such as SNOMEDCT. The alignment threshold used was 60%, meaning that matches with a similarity value below 60% were discarded. For readability reasons, we present the outcomes of our evaluation for three ontologies: SNOMEDCT, ICD9CM and CIDO. We compared the metric *changeMatcher* (see Eq. 3) between the default matcher and AML. Note that we evaluated only the difference in heuristic changes, since we observed that the impact is less sensible for the other types of changes. In Fig. 11, we can observe the significant impact of AML on one diff computation of CIDO and one SNOMEDCT computation. These diffs have few heuristic changes, thus a small variation in the quantity of detected changes significantly increases the outcome of the metrics that we used. Otherwise, AML has a positive impact on the detection of changes in SNOMEDCT, allowing us to detect (on average) more cases of heuristic changes than our built-in matcher. The impact was less impressive in the other types of ontologies. The reason for this is the complexity of the relations within the ontology; the mappings between triples are more complex to determine, requiring more efficient matchers. Another factor to consider is the quantity of changes between versions. A matcher can increase the execution time of the algorithm. Thus, it is necessary to balance the efficiency of a matcher, the quantity of heuristic changes and the execution time of the algorithm.

To better understand how matchers affect the execution time, we recommend looking at the benchmarks provided by the OAEI. In our case, we used the 2019 benchmark [36] to select AML. When using DynDiff, the end-users can select the most adapted matcher for their applied scenario.

## Conclusion

Computing ontology diff in a scalable and efficient manner is still a problem, especially for the ever-growing size of ontologies. In this paper, we presented DynDiffOnto, an ontology that defines a balanced set of change actions to support the interpretation of how ontologies evolve. We also present the DynDiff tool designed to compute the Diff between versions of ontologies and classify them according to DynDiffOnto. Our analysis shows that DynDiff is efficient for handling large ontologies, even those with dynamic instances and properties. Finally, we compared our approach with the state-of-the-art tools used in this field and observed a very close performance in terms of execution time and ability to identify changes. This tool was evaluated with ontologies from the medical domain and the importance of wisely selecting the matcher was also discussed and evaluated. The impact of blank nodes in the diff calculation is still an open question and is part of the perspectives of this work. The future of this tool now depends on Dynaccurate^9^, a spinoff that took over the development and commercialization of this technology.

## Data Availability

The datasets generated and/or analysed during the current study are available in the Bioportal repository, https://bioportal.bioontology.org.

## Footnotes

1 This paper is an extended version of [24]

2 https://protege.stanford.edu

3 http://oaei.ontologymatching.org/

4 https://git.list.lu/dynaccurate/change-ontology/-/blob/master/Comparison.pdf

5 https://jena.apache.org/documentation/fuseki2/

6 https://spark.apache.org/docs/latest/index.html

7 https://github.com/AgreementMakerLight/AML-Project/releases/tag/v3.1

8 https://github.com/AgreementMakerLight/AML-Project/releases/tag/v3.1

9 https://www.dynaccurate.com/
